# Supplementary material for: Electrochemical Performance Enhancement of Micro-Sized Porous Si by Integrating with Nano-Sn and Carbonaceous Materials
Source: Materials (Basel). 2021 Feb 15;14(4):920. doi: 10.3390/ma14040920 (PMC7919461; doi:10.3390/ma14040920)
Supplement: Supplementary file 1 [file materials-14-00920-s001.pdf]

Supporting Information

Electrochemical performance enhancement of micro-sized porous Si by integrating with nano-Sn and carbonaceous materials

Tiantian Yang [1], Hangjun Ying [1,*], Shunlong Zhang [1], Jianli Wang [1], Zhao Zhang [1] and Wei-Qiang Han [1,*]

[1] School of Materials Science and Engineering, Zhejiang University, Hangzhou, 310027, PR China; dayday_y@foxmail.com; zhangshunlong@zju.edu.cn; 11726038@zju.edu.cn; 11826056@zju.edu.cn;
* Correspondence: yinghangjun@zju.edu.cn; hanwq@zju.edu.cn

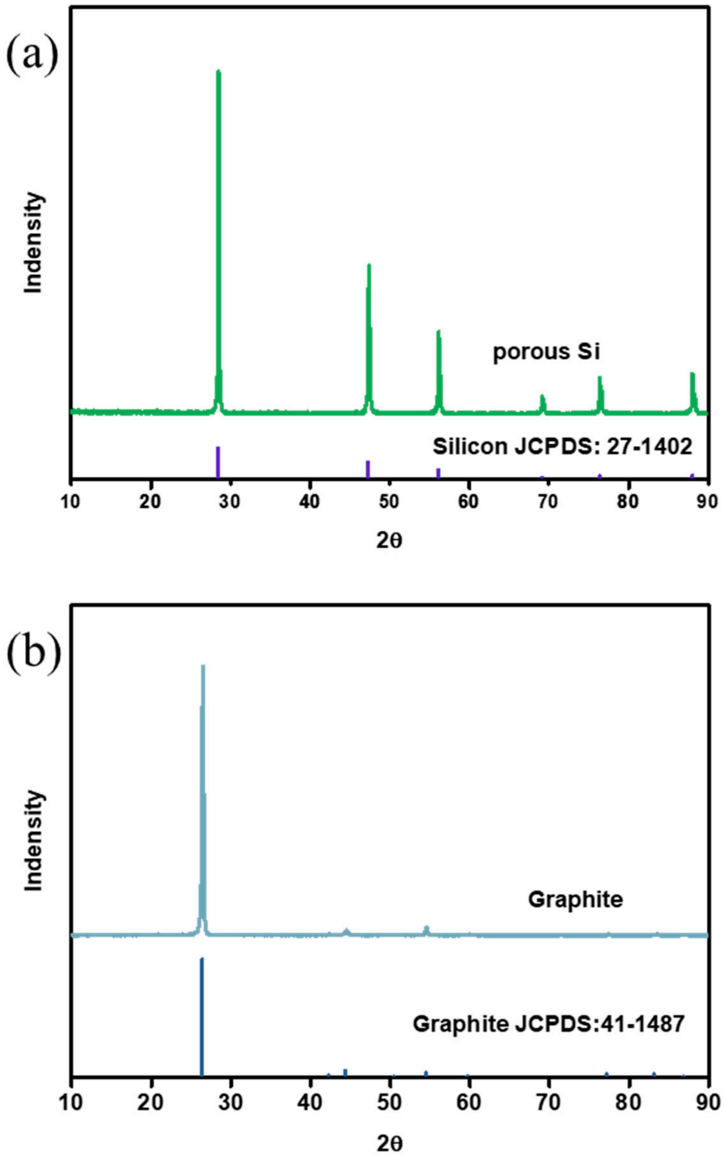

Figure S1. XRD pattern of (a) porous Si, (b) graphite.

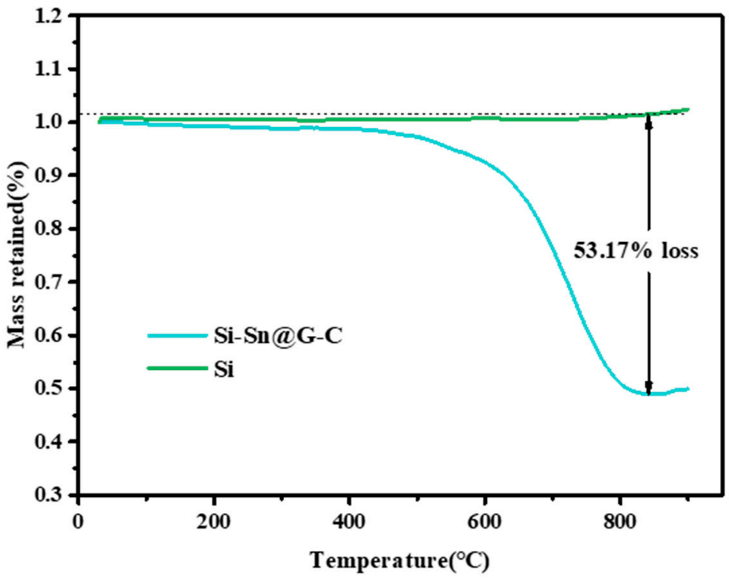

Figure S2. TGA curve of Si-Sn@G-C composite.

Table S1. Comparison of electrochemical performance of Si based anodes in literature.

Electrode	Si source	ICE	Highest capacitances obtained (mAh g^{-1})	Capacity retention	Ref
Si/graphite	Si powder (nano-sized)	77%	1001 mAh g^{-1} at 0.1 A g^{-1}	80% capacity retention over 100 cycles	[1]
Silicon sponge	Si wafer	56%	790 mAh g^{-1} at 0.1 A g^{-1}	~92.0% capacity retention over 300 cycles	[2]
Si/C composite	Si powder (1-2 μm, 99.99%)	82%	1860 mAh g^{-1} at 0.1 A g^{-1}	68% capacity retention over 60 cycles	[3]
Si/C composite	Al-Si alloy ingot	61%	952 mAh g^{-1} at 0.2 A g^{-1}	86.8% capacity retention over 300 cycles	[4]
Si/graphite/pyrolytic carbon (SiGC)	micro-sized Si powder	>80%	818 at 0.1 A/g	83.6% capacity retention over 300 cycles	[5]
Porous C-Si	$SiCl_4$	88%	2820 mAh g^{-1} at 0.4 A g^{-1}	99% capacity retention over 100 cycles	[6]
(Si-SiO-SiO_2)-C composite	SiO (325 mesh)	80%	1280 mAh g^{-1} at 0.2 A g-1	99.5% capacity retention over 200 cycles	[7]
Si/Sn@C-G	Si powder (nano-sized)	81%	1022 mAh g^{-1} at 0.1 A g^{-1}	60% capacity retention over 100 cycles	[8]
Si-Sn-DHCNFs(double-holed carbon nanofibers)	Si powder (nano-sized)	66%	1074 mAh g^{-1} at 0.1 A g^{-1}	54% capacity retention over 31 cycles	[9]
Si/Sn composites	SiSnAl alloy	76%	2466 mAh g^{-1} at 0.2 A g^{-1}	63% capacity retention over 70 cycles	[10]
Si/Sn@G-C(as prepared)	Fe-Si alloy powder	79%	1227 mAh g^{-1} at 1 A g^{-1}	96% capacity retention over 100 cycles	as prepared

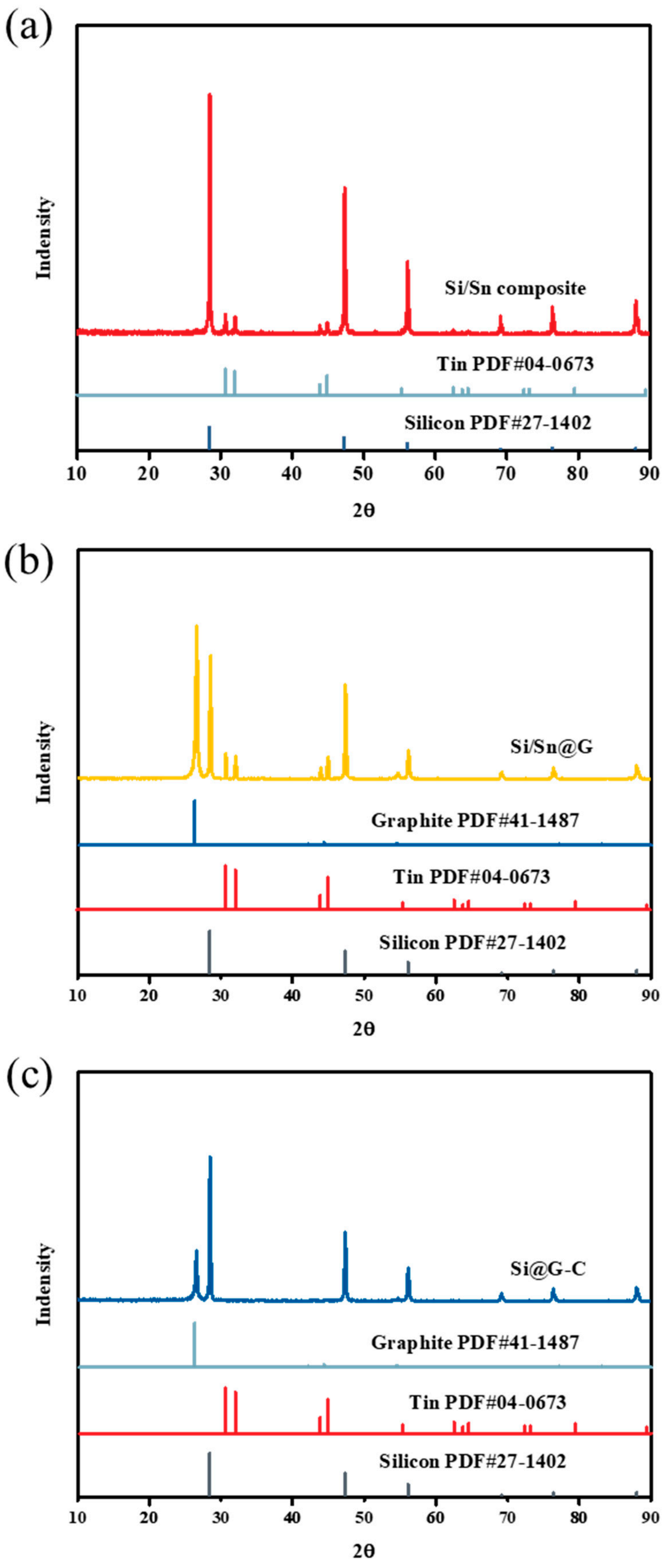

Figure S3. XRD pattern of (a) Si-Sn composite (b) Si/Sn@G composite and (c) Si@G-C composite.

Table S2. The corresponding elemental contents of the EDS of Si/Sn@G-C composite in Figure 2f-i.

Element	wt%	atom%
C	80.88	88.85
Si	9.41	4.43
Sn	1.80	0.20
O	7.91	6.52

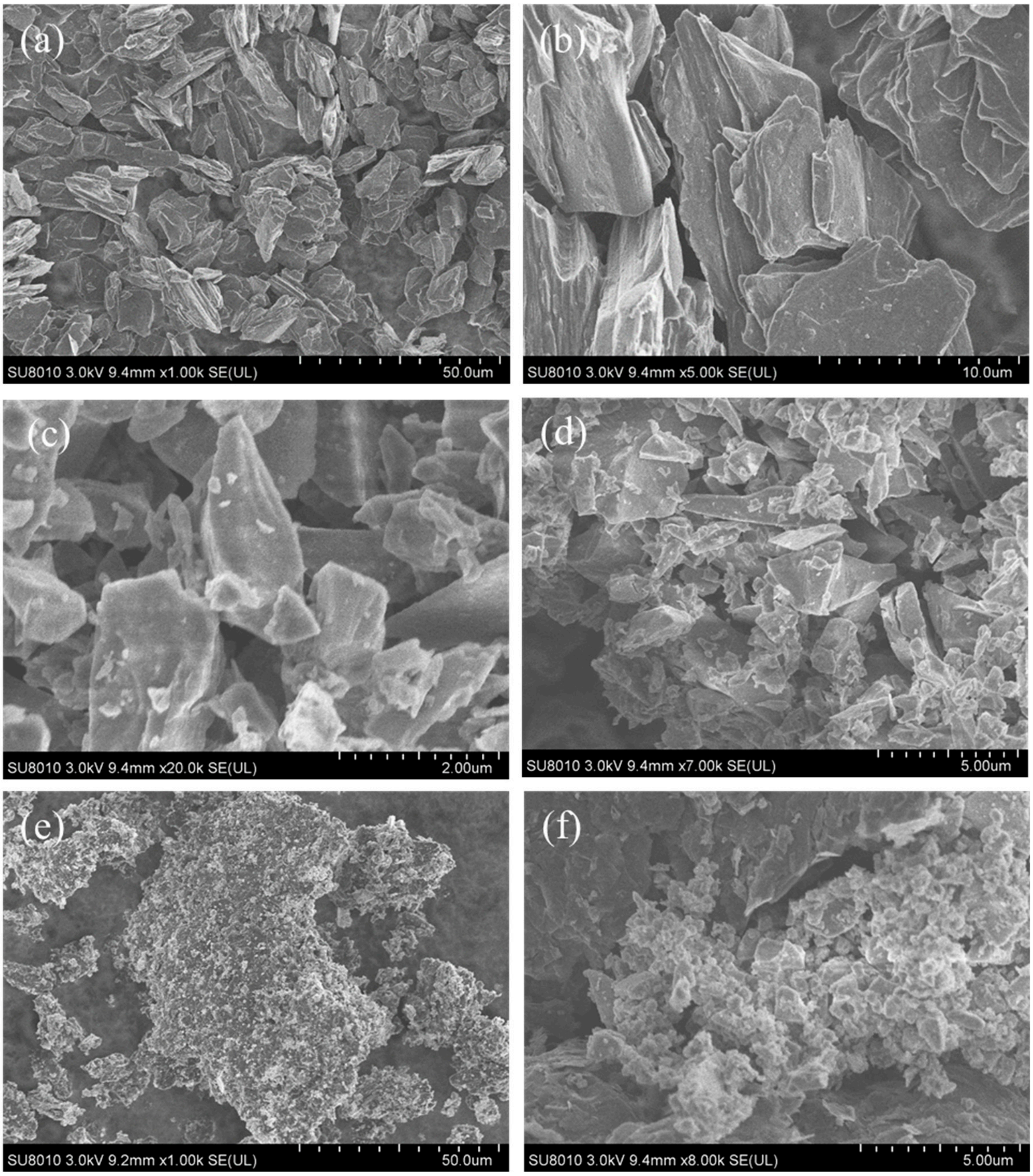

Figure S4. Top-down SEM of (a, b) the graphite (c, d) porous Si (e) Si@G-C composite, (f) Si/Sn@G composite.

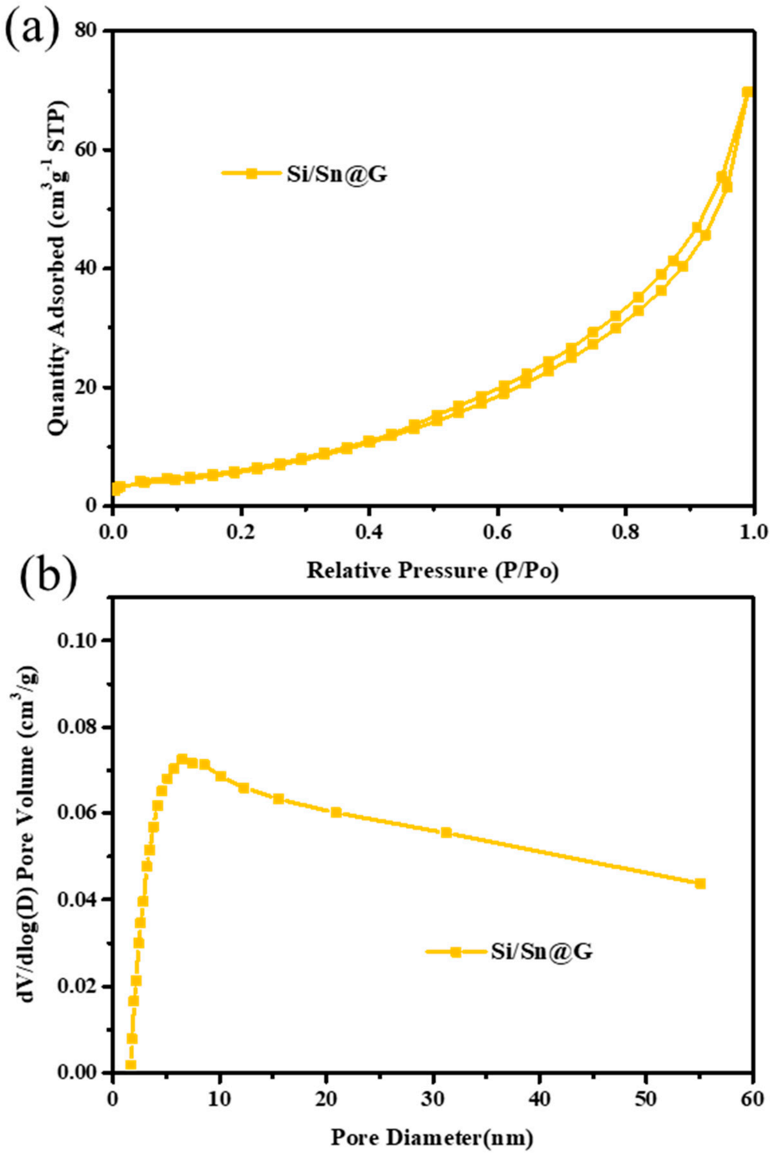

Figure S5. The BET results of the Si/Sn@G (a,) The N_2 absorption and desorption curves of the composites and (b) the corresponding pore size distribution.

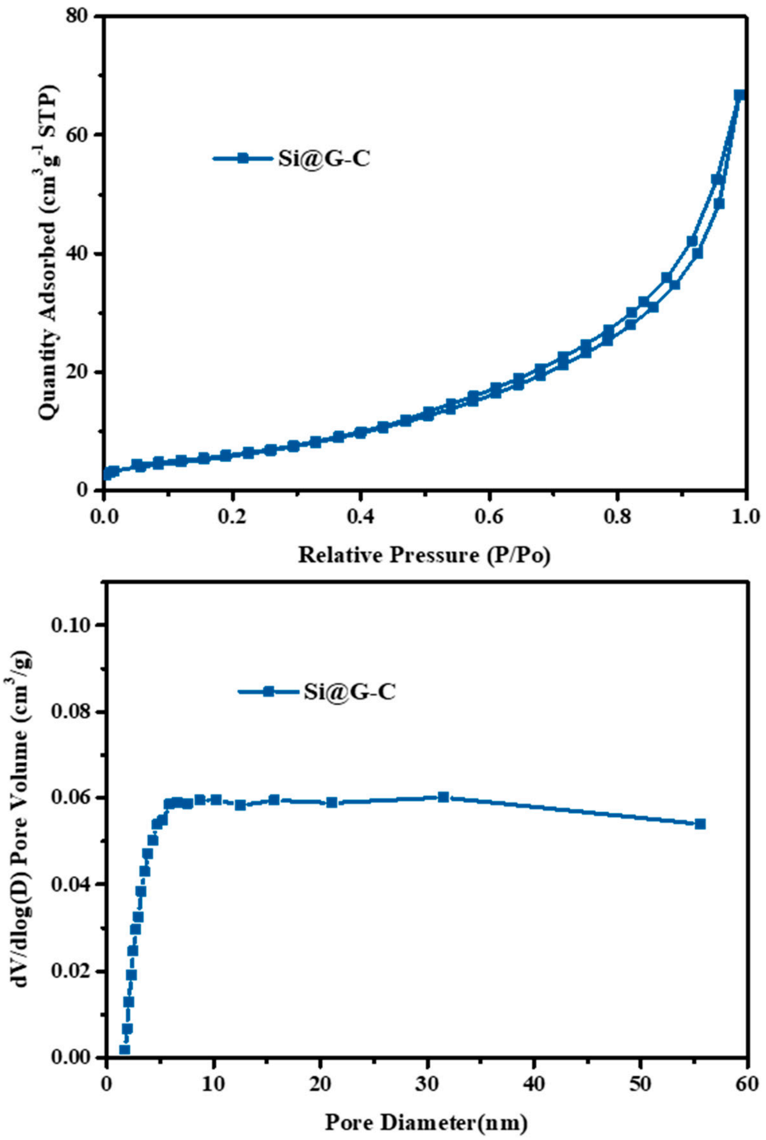

Figure S6. The BET results of the Si @G-C (a,) The N_2 absorption and desorption curves of the composites and (b) the corresponding pore size distribution.

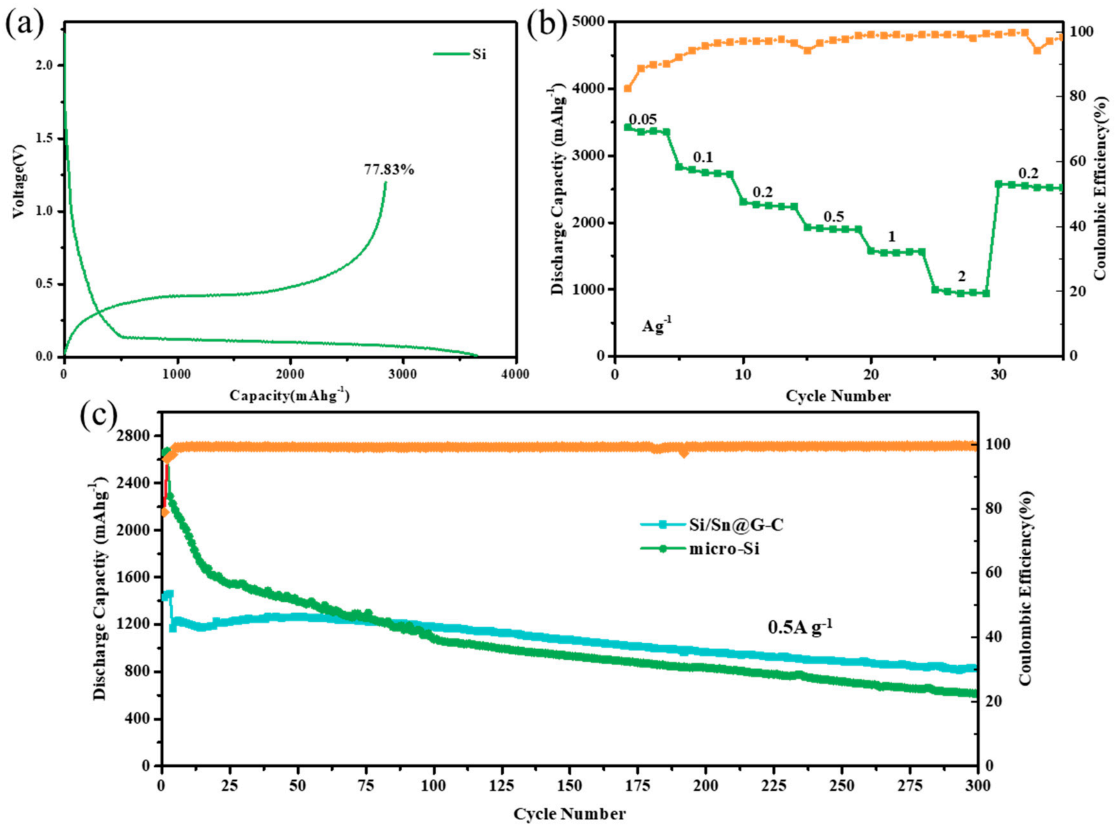

Figure S7. Electrochemical of micro sized porous Si (a) the charge/discharge profiles (b) the rate performance (c) the cycle performance

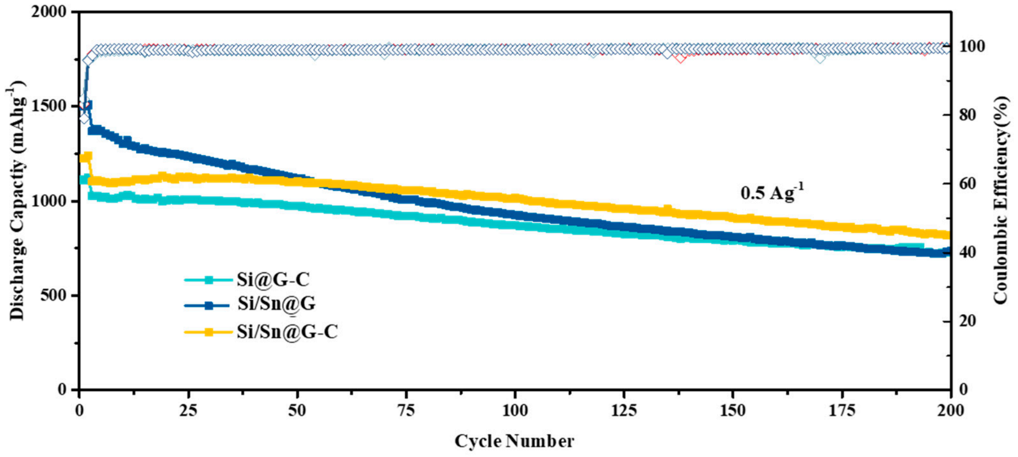

Figure S8. cycling performance at a current density of $0.5Ag^{-1}$ of Si/Sn@G-C, Si@G-C and Si/Sn@G anode.

References

1. Cabello, M.; Gucciardi, E.; Herran, A.; Carriazo, D.; Villaverde, A.; Rojo, T. Towards a High-Power Si@graphite Anode for Lithium Ion Batteries through a Wet Ball Milling Process. *Molecules* **2020**, *25*, doi:10.3390/molecules25112494.
2. Li, X.; Gu, M.; Hu, S.; Kennard, R.; Yan, P.; Chen, X.; Wang, C.; Sailor, M.J.; Zhang, J.G.; Liu, J. Mesoporous silicon sponge as an anti-pulverization structure for high-performance lithium-ion battery anodes. *Nat. Commun.* **2014**, *5*, 4105, doi:10.1038/ncomms5105.
3. Wang, D.; Gao, M.; Pan, H.; Liu, Y.; Wang, J.; Li, S.; Ge, H. Enhanced cycle stability of micro-sized Si/C anode material with low carbon content fabricated via spray drying and in situ carbonization. *J. Alloys Compd.* **2014**, *604*, 130–136, doi:10.1016/j.jallcom.2014.03.125.

4. Tian, H.; Tan, X.; Xin, F.; Wang, C.; Han, W. Micro-sized nano-porous Si/C anodes for lithium ion batteries. *Nano Energy* **2015**, *11*, 490–499, doi:10.1016/j.nanoen.2014.11.031.
5. Sui, D.; Xie, Y.; Zhao, W.; Zhang, H.; Zhou, Y.; Qin, X.; Ma, Y.; Yang, Y.; Chen, Y. A high-performance ternary Si composite anode material with crystal graphite core and amorphous carbon shell. *J. Power Sources* **2018**, *384*, 328–333, doi:10.1016/j.jpowsour.2018.03.008.
6. Kim, H.; Han, B.; Choo, J.; Cho, J. Three-dimensional porous silicon particles for use in high-performance lithium secondary batteries. *Angew Chem Int Ed Engl* **2008**, *47*, 10151–10154, doi:10.1002/anie.200804355.
7. Lee, J.-I.; Choi, N.-S.; Park, S. Highly stable Si-based multicomponent anodes for practical use in lithium-ion batteries. *Energy Environ. Sci.* **2012**, *5*, doi:10.1039/c2ee21380j.
8. Yang, D.; Shi, J.; Shi, J.; Yang, H. Simple synthesis of Si/Sn@C-G anodes with enhanced electrochemical properties for Li-ion batteries. *Electrochim. Acta* **2018**, *259*, 1081–1088, doi:10.1016/j.electacta.2017.10.117.
9. Lee, B.-S.; Yang, H.-S.; Lee, K.H.; Han, S.; Yu, W.-R. Rational design of a Si-Sn-C ternary anode having exceptional rate performance. *Energy Storage Mater.* **2019**, *17*, 62–69, doi:10.1016/j.ensm.2018.08.001.
10. Hao, Q.; Hou, J.; Ye, J.; Yang, H.; Du, J.; Xu, C. Hierarchical macroporous Si/Sn composite: Easy preparation and optimized performances towards lithium storage. *Electrochim. Acta* **2019**, *306*, 427–436, doi:10.1016/j.electacta.2019.03.163.
